# Genetic diversity within dominant *Enterocytozoon bieneusi* genotypes in pre-weaned calves

**DOI:** 10.1186/s13071-018-2768-x

**Published:** 2018-03-12

**Authors:** Chuanxiang Tang, Min Cai, Lin Wang, Yaqiong Guo, Na Li, Yaoyu Feng, Lihua Xiao

**Affiliations:** 1 State Key Laboratory of Bioreactor Engineering, School of Resource and Environmental, East China University of Science and Technology, Shanghai, 200237 China; 2 Key Laboratory of Zoonosis of Ministry of Agriculture, College of Veterinary Medicine, South China Agricultural University, Guangzhou, 510642 China; 3 Eco-environmental Protection Research Institute, Shanghai Academy of Agricultural Sciences, Shanghai, 201403 China; 4 Division of Foodborne, Waterborne, and Environmental Diseases, National Center for Emerging and Zoonotic Infectious Diseases, Centers for Disease Control and Prevention, Atlanta, GA 30333 USA

**Keywords:** *Enterocytozoon bieneusi*, Transmission, Dairy calves, Genetic diversity, Multilocus sequence typing

## Abstract

**Background:**

Cattle are commonly infected with the microsporidian parasite *Enterocytozoon bieneusi*. Sequence characterization of *E. bieneusi* in these animals at the ribosomal internal transcribed spacer (ITS) locus had identified I, J and BEB4 as the dominant genotypes. However, current studies on *E. bieneusi* in dairy cattle are mostly on infection rates and genotype distribution. This study aims to examine the intragenotypic diversity within dominant *E. bieneusi* genotypes in pre-weaned dairy calves in Shanghai, China.

**Methods:**

*Enterocytozoon bieneusi* genotypes and subtypes were identified by PCR sequence analysis of ITS and multilocus sequence typing (MLST), based on material from farms. Chi-square test was used to examine differences in *E. bieneusi* infection rates between farms or age groups.

**Results:**

The overall infection rate of *E. bieneusi* was 26.5% (214/809), ranging from 12.6% (Farm 5) to 38.5% (Farm 4). Infection rates increased with age during early life, with the peak infection rate (43.0%; 43/100) occurring at six weeks. Four genotypes were present, including J (*n* = 145, 67.8%), BEB4 (*n* = 59, 27.6%), CHN4 (*n* = 4, 1.9%) and CHN15 (*n* = 1, 0.5%), with the former two belonging to Group 2 and the latter two belonging to Group 1. Differences were detected in the distribution of the dominant genotypes J and BEB4 among five study farms. Altogether, 10 multilocus genotypes (MLGs) were identified in the two dominant ITS genotypes, including MLG-J1 to MLG-J8 of genotype J and MLG-B1 to MLG-B2 of genotype BEB4. MLG-B1 and MLG-B2 were recovered in Farms 1, 2 and 5, whereas MLG-J1 to MLG-J5 and MLG-J6 to MLG-J8 were found in Farms 3 and 4, respectively.

**Conclusions:**

There is extensive genetic heterogeneity within the dominant *E. bieneusi* genotypes J and BEB4 in dairy calves in Shanghai, China, and MLST should be used in molecular epidemiological studies of *E. bieneusi* in cattle.

## Background

Microsporidia are obligate intracellular parasites with a wide range of vertebrate and invertebrate hosts such as humans, farm and companion animals, and wildlife [1, 2]. Of approximately 17 human-pathogenic microsporidian species, *Enterocytozoon bieneusi* is the most frequently detected [2, 3]. In immunocompromised patients (HIV-positive patients or organ transplant recipients), *E. bieneusi* usually causes chronic diarrhea and wasting syndrome [3–5], but in immunocompetent humans and animals, *E. bieneusi* infection can be asymptomatic [6, 7].

Based on sequence analysis of the internal transcribed spacer (ITS) of the rRNA gene (~243 bp), more than 200 *E. bieneusi* genotypes have been identified [1, 8]. Phylogenetic analyses revealed that they belong to nine groups [9, 10]. Group 1, which contains most genotypes found in humans, is considered a zoonotic group, with the remaining groups being largely host-specific. To date, over 40 *E. bieneusi* genotypes have been detected in cattle, most of which belong to Group 2 [11–13]. Among them, at least 15 genotypes, including eight genotypes in Group 1 and seven genotypes in Group 2, have been reported in humans [11, 12, 14], suggesting that cattle may be potential reservoirs for human infections.

Genotypes I, J and BEB4 are common *E. bieneusi* genotypes found in pre-weaned dairy calves worldwide [8, 11, 12, 15–21] and have been further detected in at least 13 human cases [6, 22]. However, current studies on *E. bieneusi* in dairy calves are mostly on infection rates and genotype distribution. Little is known about the age distribution of *E. bieneusi* infection in pre-weaned dairy calves. In addition, genetic diversity within the dominant *E. bieneusi* genotypes has not been examined thoroughly using advanced molecular diagnostic tools such as multilocus sequence typing (MLST).

MLST has been used in investigations of *E. bieneusi* transmission in humans [23, 24], non-human primates [25–27], giant pandas [26, 28, 29], red pandas [26, 28], bears [26], lions [26], golden cats [26], deer [26], alpacas [26], blackbucks [26], raccoons [26], golden takins [30], horses [31], raccoon dogs [32], foxes [32, 33] and red-bellied tree squirrels [34]. Thus far, there has been only one study on multilocus characterization of *E. bieneusi* in cattle in Shaanxi, China, and the data were not analyzed for intra-genotypic variations and transmission among farms [17]. In this study, MLST was used to assess genetic heterogeneity within dominant *E. bieneusi* genotypes of Group 2 in pre-weaned calves, and the age pattern of *E. bieneusi* infection during early life of cattle was examined.

## Methods

### Specimen collection

From April 2015 to March 2016, 809 specimens, each of approximately 25 g fresh fecal material, were collected from pre-weaned Holstein calves in five farms in Shanghai, China. These farms are located in Fengxian (Farms 1, 2, 3 and 4) and Jinshan (Farm 5), two neighboring districts in suburban Shanghai. They were ranked A to E by combined farm quality score based on hygiene status, animal density, and facility condition, with A representing “excellent” and E representing “poor” [35]. Each farm was visited 2–5 times at 2–3 months intervals, for a total of five times for Farm 3, four times for Farm 1, and twice for Farms 2, 4 and 5. These fecal specimens were collected directly from the rectum by using disposable gloves into 50 ml centrifuge tubes, transported to the laboratory in coolers with ice packs, and stored in 2.5% potassium dichromate at 4 °C before DNA extraction.

### DNA extraction

Genomic DNA was extracted by using the Fast DNA SPIN Kit for soil (MP Biomedical, Santa Ana, CA, USA) from approximately 200 mg of each fecal specimen, which was washed three times with distilled water by centrifugation at 2000× *g* for 10 min. The obtained DNA was stored at -20 °C until being used in PCR analysis.

### PCR analysis

The occurrence and genotype distribution of *E. bieneusi* were determined by PCR and sequence analyses of the ITS as previously described [36]. For subtyping the dominant *E. bieneusi* ITS genotypes J and BEB4, the MLST technique targeting microsatellite loci MS1, MS3 and MS7 and minisatellite locus MS4 was used [24]. In a pre-study analysis, 90 of 98 *E. bieneusi-*positive specimens yielded the expected PCR products at the MS3 locus. Therefore, PCR analysis of the MS3 locus was used for screening of the 204 specimens positive for ITS genotypes J and BEB4. Among them, 84 MS3-positive specimens of five different MS3 subtypes were further analyzed at the MS1, MS7 and MS4 loci. Duplicate nested PCR was used in the analysis of the specimens at each genetic locus. The secondary PCR products obtained were identified by agarose gel electrophoresis.

### Sequence analysis

All secondary PCR products of the expected size were bi-directionally sequenced using the secondary PCR primers on an ABI 3730 Genetic Analyzer (Applied Biosystems, Foster City, CA, USA). The obtained sequences were assembled using ChromasPro 2.1.5.0 (http://technelysium.com.au/ChromasPro.html), edited manually for sequence miscalls using BioEdit 7.1.3.0 (http://www.mbio.ncsu.edu/BioEdit/bioedit.html), and aligned with reference sequences from the GenBank database using ClustalX 2.0.11 (http://clustal.org). Only sequence data from specimens that were successfully subtyped at all four MLST loci were used in the determination of multilocus genotypes (MLGs). MLGs were named according to the ITS genotypes: MLG-J1 to MLG-J8 for ITS genotype J and MLG-B1 to MLG-B2 for ITS genotype BEB4.

### Statistical analysis

Differences in *E. bieneusi* infection rates between farms or age groups were examined by using the Chi-square test implemented in SPSS Statistics v.20.0 for Windows (IBM Corp., New York, NY, USA). Differences were considered significant at *P* ≤ 0.05.

## Results

### Occurrence of *E. bieneusi* in pre-weaned dairy calves

Among the 809 specimens collected from pre-weaned calves in five farms, 214 (26.5%) were positive for *E. bieneusi* in PCR analysis of the ITS locus. All five farms had *E. bieneusi*, with infection rates ranging between 12.6–38.5%. The highest infection rate (38.5%; 15/39) was in Farm 4, while the lowest (12.6%; 26/206) was in Farm 5 (Table 1). Farms with good management (such as Farms 2 and 5) had lower *E. bieneusi* infection rates than farms with relatively poor management (such as Farm 4). In the former, the infection rates were 16.1% (9/56) on Farm 2 and 12.6% (26/206) on Farm 5, whereas in the latter the infection rate was 38.5% (15/39) on Farm 4 (*χ*^2^ = 6.104, *df* = 1, *P* = 0.013 between Farms 2 and 4; and *χ*^2^ = 15.714, *df* = 1, *P* < 0.0001 between Farms 5 and 4).

**Table 1:** Occurrence and distribution of *Enterocytozoon bieneusi* ITS genotypes and multilocus genotypes (MLGs) in pre-weaned dairy calves on five farms in Shanghai, China

Farm	Farm rank^a^	Sampling point	Sample size	No. positive for *E. bieneusi* (%)	ITS genotype (No.)	MLG (No.)
1	D	1	36	12 (33.3)	BEB4 (11), Mixed infection (1)	MLG-B1 (2)
		2	12	3 (25.0)	BEB4 (3)	–
		3	46	13 (28.3)	BEB4 (13)	MLG-B1 (2), MLG-B2 (1)
		4	25	5 (20.0)	BEB4 (5)	MLG-B2 (1)
Subtotal			119	33 (27.7)	BEB4 (32), Mixed infection (1)	MLG-B1 (4), MLG-B2 (2)
2	A	1	47	6 (12.8)	CHN4 (4), Type IV and BEB4 (2)	–
		2	9	3 (33.3)	BEB4 (1), Type IV and BEB4 (2)	MLG-B1 (1)
Subtotal			56	9 (16.1)	CHN4 (4), BEB4 (1), Type IV and BEB4 (4)	MLG-B1 (1)
3	B	1	112	44 (39.3)	J (42), BEB4 (1), CHN15 (1)	MLG-J1 (1), MLG-J2 (1), MLG-J4 (1)
		2	43	17 (39.5)	J (17)	MLG-J3 (1), MLG-J5 (1)
		3	81	24 (29.6)	J (24)	MLG-J2 (4)
		4	84	19 (22.6)	J (19)	MLG-J2 (1)
		5	69	27 (39.1)	J (27)	MLG-J2 (4)
Subtotal			389	131 (33.7)	J (129), BEB4 (1), CHN15 (1)	MLG-J2 (10), MLG-J1 (1), MLG-J3 (1), MLG-J4 (1), MLG-J5 (1)
4	E	1	29	12 (41.4)	J (12)	MLG-J6 (3), MLG-J7 (1), MLG-J8 (1)
		2	10	3 (30.0)	J (3)	MLG-J6 (1)
Subtotal			39	15 (38.5)	J (15)	MLG-J6 (4), MLG-J7 (1), MLG-J8 (1)
5	C	1	109	21 (19.3)	BEB4 (20), J (1)	MLG-B1 (7), MLG-B2 (1)
		2	97	5 (5.2)	BEB4 (5)	–
Subtotal			206	26 (12.6)	BEB4 (25), J (1)	MLG-B1 (7), MLG-B2 (1)
Total			809	214 (26.5)	J (145), BEB4 (59), CHN4 (4), Type IV and BEB4 (4), CHN15 (1), Mixed infection (1)	MLG-B1 (12), MLG-J2 (10), MLG-J6 (4), MLG-B2 (3), MLG-J1 (1), MLG-J3 (1), MLG-J4 (1), MLG-J5 (1), MLG-J7 (1), MLG-J8 (1)

^a^Farm ranks A-E were ranking scores used to evaluate the hygiene status, animal density and facility condition, with A representing “excellent” and E representing “very poor” (see [35] for details)

### Age distribution of *E. bieneusi* infection

**Fig. 1:**
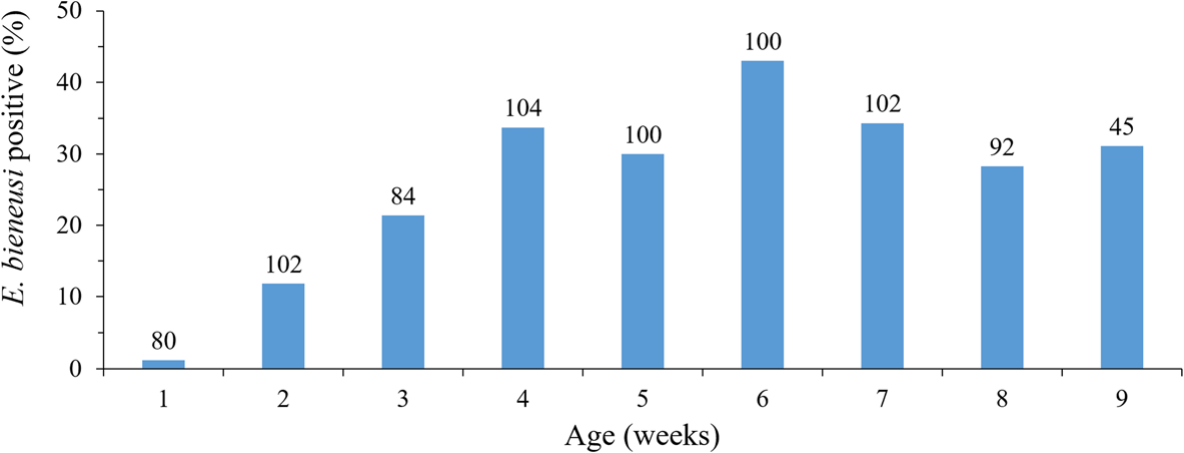
Occurrence of *Enterocytozoon bieneusi* in pre-weaned dairy calves on five farms in Shanghai, China by age. The numbers above columns are numbers of specimens analyzed per age group

By age in weeks, *E. bieneusi* infection rates increased gradually during the first four weeks of life, with the highest infection rate (43.0%; 43/100) reached at six weeks (Fig. 1). The overall infection rate at 4–7 weeks of age was 35.2% (143/406), which was significantly higher than the infection rate of 11.7% (31/266) at 1–3 weeks of age (*χ*^2^ = 46.519, *df* = 1, *P* < 0.0001).

### *Enterocytozoon bieneusi* infection and occurrence of diarrhea

The specimens in this study were from three groups of animals: calves with watery diarrhea (G1, *n* = 85), moderate diarrhea (G2, *n* = 346), or no diarrhea (G3, *n* = 378). G1 specimens had a slightly higher *E. bieneusi* infection rate (30.6% or 26/85) than G2 (25.1% or 87/346) and G3 (26.7% or 101/378) specimens. The differences among the three groups were not significant (*χ*^2^ = 0.522, *df* = 1, *P* = 0.470 between G1 and G3; and *χ*^2^ = 0.233, *df* = 1, *P* = 0.629 between G2 and G3).

### ITS genotypes of *E. bieneusi* by farm

DNA sequencing of ITS PCR products was successful for 209 of 214 PCR-positive specimens. The remaining five specimens generated ITS sequences with underlying signals because of the presence of mixed *E. bieneusi* genotypes. Four *E. bieneusi* genotypes were identified among the 209 successfully genotyped specimens, including J, BEB4, CHN4 and CHN15, with the latter being identical to an unnamed genotype (JF909995) in wastewater from Tunisia [37]. The dominant genotypes in calves were J (*n* = 145, 67.8%) and BEB4 (*n* = 59, 27.6%), which both belong to Group 2. The remaining two genotypes, CHN4 and CHN15, were seen in only four (1.9%) and one (0.5%) *E. bieneusi*-positive calves, respectively (Table 1). Among the five farms, Farms 1 and 4 each had only one genotype, whereas Farms 2, 3, and 5 each had two or three genotypes. The dominant genotype in farms with higher infection rates (Farms 3 and 4) was genotype J, compared with genotype BEB4 in farms with lower infection rates (Farms 1 and 5). Among the five specimens with mixed *E. bieneusi* genotypes, four from Farm 2 had concurrence of genotypes Type IV and BEB4.

### Distribution of MLST subtypes

MLST analysis was conducted on 84 specimens of the two dominant ITS genotypes J (Farms 3 and 4) and BEB4 (Farms 1, 2 and 5) to assess intra-genotypic variations in pre-weaned dairy calves, after screening all 204 specimens that were positive for the two ITS genotypes by using MS3 PCR. The overall amplification efficiency at the MS1, MS3, MS4 and MS7 loci was 92.9% (78/84), 69.6% (142/204), 94.0% (79/84) and 41.7% (35/84), respectively (Table 2). The amplification efficiency of ITS genotypes J and BEB4 was similar at the MS1 (92.5 and 93.5%, respectively), MS4 (92.5 and 96.8%, respectively) and MS7 (37.7 and 48.4%, respectively) loci. However, at the MS3 locus, there was an obvious difference (58.6 *vs* 96.6%) in amplification efficiency between these two dominant genotypes. In addition, all 15 genotype J-positive specimens from Farm 4 were negative in MS3 PCR, while those from Farm 3 produced the expected MS3 PCR products.

**Table 2:** PCR amplification efficiency of DNA from *Enterocytozoon bieneusi* ITS genotypes J and BEB4 at the MLST loci

ITS genotype	Farm ID	No. of specimens	No. of specimens amplified/No. of specimens analyzed			
			MS1	MS3	MS4	MS7
J	1	0	–	–	–	–
	2	0	–	–	–	–
	3	129	35/38	85/129	35/38	14/38
	4	15	14/15	0/15	14/15	6/15
	5	1	–	0/1	–	–
BEB4	1	32	13/15	31/32	14/15	6/15
	2	1	1/1	1/1	1/1	1/1
	3	1	–	0/1	–	–
	4	0	–	–	–	–
	5	25	15/15	25/25	15/15	8/15
Total		204	78/84	142/204	79/84	35/84

Altogether, there were eight, five, two and four subtypes at the MS1, MS3, MS4 and MS7 loci, respectively. The diversity of subtype was different between genotypes J and BEB4, with seven, five, two, and three subtypes being detected in genotype J, compared to one, one, one, and two subtypes in BEB4, respectively.

As expected, the dominant subtype at each locus was different between genotypes J and BEB4 (Table 3). At the MS1 locus, the dominant subtype was MS1-3 in genotype J (28/53), while it was MS1-1 in BEB4 (29/31). At the MS3 locus, the dominant subtype was MS3-1 in genotype J (29/53), while it was MS3-2 in BEB4 (31/31). At the MS4 locus, MS4-1 (24/53) and MS4-2 (24/53) were the dominant subtypes in genotype J, while MS4-2 was the only subtype in BEB4 (30/31). At the MS7 locus, the dominant subtype was MS7-1 both in genotypes J (18/53) and BEB4 (12/31).

**Table 3:** Occurrence and distribution of subtypes from *Enterocytozoon bieneusi* ITS genotypes J and BEB4 at four MLST loci

ITS genotype	Farm ID	Subtype				No. of positive specimens
		MS1	MS3	MS4	MS7	
J	3	MS1-3^n^	MS3-1	MS4-2	–	12
		MS1-3^n^	MS3-1	MS4-2	MS7-1	10
		MS1-3^n^	MS3-1	MS4-1	–	1
		MS1-3^n^	MS3-1	–	–	1
		MS1-3^n^	MS3-1	MS4-1	MS7-1	1
		MS1-3^n^	MS3-1	MS4-2 and MS4-3	–	1
		MS1-3^n^	MS3-2	MS4-1	–	1
		MS1-3^n^	MS3-4^n^	MS4-2	MS7-1	1
		MS1-2	MS3-2	MS4-1	–	1
		MS1-2	–	–	–	1
		MS1-4^n^	MS3-3^n^	MS4-1	–	1
		MS1-5^n^	MS3-2	MS4-1	MS7-1	1
		MS1-5^n^	MS3-5^n^	MS4-1	MS7-1	1
		MS1-6^n^	MS3-5^n^	MS4-1	–	1
		MS1-7^n^	MS3-1	MS4-1	–	1
		–	MS3-1	MS4-2	–	1
		–	MS3-1	–	–	1
		–	MS3-2	MS4-1	–	1
	4	MS1-8^n^	–	MS4-1	–	6
		MS1-8^n^	–	MS4-1	MS7-1	4
		MS1-8^n^	–	MS4-1	MS7-2^n^	1
		MS1-8^n^	–	MS4-1	MS7-4^n^	1
		MS1-8^n^	–	–	–	1
		MS1-7^n^	–	MS4-1	–	1
		Noisy	–	MS4-1	–	1
BEB4	1	MS1-1	MS3-2	MS4-2	–	7
		MS1-1	MS3-2	MS4-2	MS7-1	4
		MS1-1	MS3-2	MS4-2	MS7-3^n^	2
		–	MS3-2	MS4-2	–	1
		–	MS3-2	–	–	1
	2	MS1-1	MS3-2	MS4-2	MS7-1	1
	5	MS1-1	MS3-2	MS4-2	MS7-1	7
		MS1-1	MS3-2	MS4-2	–	7
		MS1-1	MS3-2	MS4-2	MS7-3^n^	1
Total						84

*Abbreviation: n* novel subtype

The distribution of subtypes in genotype J differed between Farms 3 and 4. The main subtypes on Farm 3 were MS1-3, MS3-1, MS4-2 and MS7-1 at the four loci, while the main subtypes on Farm 4 were MS1-8, no MS3 amplification, MS4-1 and MS7-1. In contrast, the main subtypes in genotype BEB4 at the four loci were the same (MS1-1, MS3-2, MS4-2 and MS7-1) on all BEB4-positive farms (Farms 1, 2 and 5).

Of the 84 specimens analyzed by MLST, only 29 were positive at all four genetic loci, with seven MLGs obtained, including five genotype J MLGs (MLG-J1 to MLG-J5) and two genotype BEB4 MLGs (MLG-B1 to MLG-B2). To compare subtype diversity of genotype J among farms, six additional specimens from Farm 4, which were successfully subtyped at MS1, MS4 and MS7 loci but were PCR-negative at the MS3 locus, were included in the MLGs analysis. They were assigned the MLG-J6 to MLG-J8 because of the likely presence of a unique MS3 sequence (Table 4). The dominant MLGs were MLG-B1 (*n* = 12) and MLG-J2 (*n* = 10). Between them, MLG-B1 was the dominant MLG in three farms (Farms 1, 2 and 5), while MLG-J2 was the predominant MLG in only one farm (Farm 3). In addition, the distribution of genotype J MLGs was different between Farms 3 (MLG-J1 to MLG-J5) and 4 (MLG-J6 to MLG-J8), whereas the distribution of genotype BEB4 MLGs was similar among Farms 1 (MLG-B1 and MLG-B2), 2 (MLG-B1) and 5 (MLG-B1 and MLG-B2) (Table 1).

**Table 4:** Multilocus sequence types of *Enterocytozoon bieneusi* in pre-weaned dairy calves by ITS genotype in Shanghai, China

MLG	ITS Genotype	Multilocus type				No. of MLGs	Farm (no. of specimens)
		MS1	MS3	MS4	MS7		
J1	J	MS1-3^n^	MS3-4^n^	MS4-2	MS7-1	1	3 (1)
J2	J	MS1-3^n^	MS3-1	MS4-2	MS7-1	10	3 (10)
J3	J	MS1-3^n^	MS3-1	MS4-1	MS7-1	1	3 (1)
J4	J	MS1-5^n^	MS3-5^n^	MS4-1	MS7-1	1	3 (1)
J5	J	MS1-5^n^	MS3-2	MS4-1	MS7-1	1	3 (1)
J6	J	MS1-8^n^	–	MS4-1	MS7-1	4	4 (4)
J7	J	MS1-8^n^	–	MS4-1	MS7-2^n^	1	4 (1)
J8	J	MS1-8^n^	–	MS4-1	MS7-4^n^	1	4 (1)
B1	BEB4	MS1-1	MS3-2	MS4-2	MS7-1	12	1 (4), 2 (1), 5 (7)
B2	BEB4	MS1-1	MS3-2	MS4-2	MS7-3^n^	3	1 (2), 5 (1)
Total						35	3 (14), 5 (8), 1 (6), 4 (6), 2 (1)

*Abbreviation*: *n* novel subtype

## Discussion

In the present study, *E. bieneusi* was found in 26.5% (214/809) of pre-weaned dairy calves in Shanghai. This is similar to the infection rate of 29.3% (127/434) reported in one study in Henan and Ningxia, but higher than rates reported in other studies in Henan and Shandong (10.0% or 1/10), Heilongjiang (7.7% or 20/259), Shaanxi (19.5% or 39/200) and Xinjiang (17.7% or 42/237) in China [12, 15–17, 21]. Similar differences (3.1–35.4%) in infection rates in pre-weaned calves have been reported in studies in the USA, Brazil, Argentina and the Czech Republic [8, 11, 18–20, 38]. Variations in *E. bieneusi* infection rates in pre-weaned dairy calves among studies could be due to differences in detection methods, age and management of animals, and climate.

Pre-weaned dairy calves appear to have peak *E. bieneusi* infection around 4–7 weeks of age. In this study, although calves were infected by *E. bieneusi* from one to nine weeks of age, *E. bieneusi* occurrence in newborn animals increased gradually with age, with the peak infection rate (43.0%) being reached at six weeks of age. Thus, the mean infection rate at 4–7 weeks of age was significantly higher than at 1–3 weeks. This agrees with the result of the only other study of the age pattern of *E. bieneusi* infection in pre-weaned dairy calves in the USA [8]. Previously in China, only slightly higher *E. bieneusi* infection rates were reported in pre-weaned dairy calves than in post-weaned dairy calves: 17.7% and 15.5% in Xinjiang [16], 29.3 and 23.9% in Henan and Ningxia [15], 10.0 and 7.3% in Henan and Shandong [21], and 7.4 and 4.3% in Northeast China [12], respectively. Lumping all pre-weaned calves into one group could be responsible for the small differences in *E. bieneusi* infection rates between pre-weaned and post-weaned calves.

Four genotypes (J, BEB4, CHN4 and CHN15) were identified among 214 *E. bieneusi*-positive specimens at the ITS locus. Genotype J was the dominant one among the four genotypes and the main genotype reported in pre-weaned dairy calves worldwide [11, 12, 15–17]. Another common genotype in the study (27.6% or 59/214), BEB4, was a genotype with lower prevalence in Xinjiang (9.5% or 4/42), Heilongjiang (5.0% or 1/20), Shaanxi (2.6% or 1/39), Henan and Ningxia (2.4% or 3/127) within China. This was also the case in the USA (10.0% or 1/10), Argentina (10.0% or 1/10) and Brazil (5.3% or 1/19) [8, 11, 12, 15–17, 20]. Between the remaining two *E. bieneusi* genotypes found in the study, CHN4 was reported in cattle in Jilin, China [22], while CHN15 was reported in wastewater in Tunisia [37]. In contrast, Genotype I, a common *E. bieneusi* genotype in pre-weaned dairy calves worldwide [8, 11, 15–21], was not detected in the present study. In the present study, the distribution of the two dominant *E. bieneusi* genotypes, J and BEB4, is different among five study farms; genotype BEB4 occurred on farms with lower infection rates of *E. bieneusi* (Farms 1, 2 and 5), whereas genotype J occurred on farms with higher infection rates (Farms 3 and 4).

Results of the MLST analysis support the existence of differences in the transmission of the two dominant *E. bieneusi* ITS genotypes. All dominant subtypes in genotype BEB4 at the four individual loci, such as MS1-1, MS3-2, MS4-2 and MS7-1, and the most common MLG (MLG-B1) in genotype BEB4 were present on all farms with ITS genotype BEB4 (Farms 1, 2 and 5). In contrast, the dominant subtypes of ITS genotype J at each locus were different between Farms 3 and 4. In fact, genotype J on Farm 4 was so divergent from the one on Farm 3 at the MS3 locus that none of the 15 genotype J-positive specimens from Farm 4 generated the expected MS3 PCR product whereas 85 of the 129 genotype J-positive specimens from Farm 3 generated it. The most common genotype J MLG (MLG-J2) was only seen on Farm 3, and all other genotype J MLGs identified in this study, were exclusively present on Farm 3 or 4. Therefore, although all five farms are owned by the same dairy enterprise and located in two neighboring districts of suburban Shanghai, there is extensive genetic heterogeneity within the dominant *E. bieneusi* genotypes, especially ITS genotype J.

## Conclusions

Results of this study indicate that *E. bieneusi* infection is common in pre-weaned dairy calves in suburban Shanghai, China, with animals of 4–7 weeks of age having the highest occurrence of the pathogen. Data of MLGs among farms suggest that there are apparent differences in the distribution of dominant *E. bieneusi* genotypes among farms and extensive genetic heterogeneity within ITS genotypes. Molecular epidemiologic studies involving advanced pathogen characterization should be conducted to improve understanding of the population genetics of *E. bieneusi* in cattle and relationship among infection rates, age-associated infection patterns, genotype distribution, farm management, and transmission of the pathogen.

## Abbreviations

ITS: Internal transcribed spacer
MLGs: Multilocus genotypes
MLST: Multilocus sequence typing
PCR: Polymerase chain reaction
